# Clinical, Neurophysiological, and Genetic Predictors of Recovery in Patients With Severe Acquired Brain Injuries (PRABI): A Study Protocol for a Longitudinal Observational Study

**DOI:** 10.3389/fneur.2022.711312

**Published:** 2022-02-28

**Authors:** Bahia Hakiki, Ida Donnini, Anna Maria Romoli, Francesca Draghi, Daniela Maccanti, Antonello Grippo, Maenia Scarpino, Antonio Maiorelli, Raisa Sterpu, Tiziana Atzori, Andrea Mannini, Silvia Campagnini, Silvia Bagnoli, Assunta Ingannato, Benedetta Nacmias, Francesco De Bellis, Anna Estraneo, Valentina Carli, Eugenia Pasqualone, Angela Comanducci, Jorghe Navarro, Maria Chiara Carrozza, Claudio Macchi, Francesca Cecchi

**Affiliations:** ^1^Istituto di Ricovero e Cura a Carattere Scientifico (IRCCS) Fondazione Don Carlo Gnocchi, Firenze, Italy; ^2^The Biorobotics Institute, Scuola Superiore Sant'Anna, Pisa, Italy; ^3^Neuroscience Section, Department of Neurofarba, University of Florence, Firenze, Italy; ^4^Istituto di Ricovero e Cura a Carattere Scientifico (IRCCS) Fondazione Don Carlo Gnocchi, Milano, Italy; ^5^Department of Experimental and Clinical Medicine, University of Florence, Firenze, Italy

**Keywords:** severe acquired brain injuries, neurophysiology, genetics, disorders of consciousness, rehabilitation

## Abstract

**Background:**

Due to continuous advances in intensive care technology and neurosurgical procedures, the number of survivors from severe acquired brain injuries (sABIs) has increased considerably, raising several delicate ethical issues. The heterogeneity and complex nature of the neurological damage of sABIs make the detection of predictive factors of a better outcome very challenging. Identifying the profile of those patients with better prospects of recovery will facilitate clinical and family choices and allow to personalize rehabilitation. This paper describes a multicenter prospective study protocol, to investigate outcomes and baseline predictors or biomarkers of functional recovery, on a large Italian cohort of sABI survivors undergoing postacute rehabilitation.

**Methods:**

All patients with a diagnosis of sABI admitted to four intensive rehabilitation units (IRUs) within 4 months from the acute event, aged above 18, and providing informed consent, will be enrolled. No additional exclusion criteria will be considered. Measures will be taken at admission (T0), at three (T1) and 6 months (T2) from T0, and follow-up at 12 and 24 months from onset, including clinical and functional data, neurophysiological results, and analysis of neurogenetic biomarkers.

**Statistics:**

Advanced machine learning algorithms will be cross validated to achieve data-driven prediction models. To assess the clinical applicability of the solutions obtained, the prediction of recovery milestones will be compared to the evaluation of a multiprofessional, interdisciplinary rehabilitation team, performed within 2 weeks from admission.

**Discussion:**

Identifying the profiles of patients with a favorable prognosis would allow customization of rehabilitation strategies, to provide accurate information to the caregivers and, possibly, to optimize rehabilitation outcomes.

**Conclusions:**

The application and validation of machine learning algorithms on a comprehensive pool of clinical, genetic, and neurophysiological data can pave the way toward the implementation of tools in support of the clinical prognosis for the rehabilitation pathways of patients after sABI.

## Introduction

Severe acquired brain injury (sABI) is characterized by traumatic, anoxic, vascular, or other brain damages that cause coma for at least 24 hand frequently lead to permanent sensorial, motor, cognitive, or behavioral disabilities. The incidence rate of sABI patients is estimated between 10 and 15 new cases/100,000 persons/year and the prevalence rate between 300 and 800 cases /100,000 persons, which represents comprehensively about 150,000 persons in 2016 in Italy (1, 2).

After sABI, some patients may survive in a state of prolonged disorder of consciousness (DoC) (3), a condition that encompasses (1) unresponsive wakefulness syndrome (UWS), in which the eyes are open, but there is no evidence of voluntary behaviors and (2) minimal consciousness state (MCS), an intermediate state in which minimal, inconsistent but reproducible behavioral signs of responsiveness are present (4). Based on the complexity of patients' behavioral responses, a subcategorization of MCS patients was proposed (5); namely, “MCS minus” (MCS–) characterizes patients with intentional low-level behavior, such as visual search or localized motor response to nociceptive stimulus, and “MCS plus” (MCS+) indicates patients with high-level behavioral interactions, such as execution of simple verbal commands (5). In some cases, patients recover full consciousness, as marked by the regaining of functional and reliable “yes/no” communication (emergence from MCS, E-MCS), but they may still present severe motor and cognitive or behavioral impairments persisting lifelong.

Besides the severity of the neurological state, patients with sABI frequently present a high clinical complexity characterized by very frequent comorbidities (6) and medical complications (7), which can further impact clinical improvement and survival. Although late consciousness recovery and progression have been reported in patients with UWS or MCS (8), long-term mortality is high (42–52%) in patients with prolonged DoC (1, 2), and survivors frequently show severe to extremely severe outcomes (9).

Younger age, shorter postevent time, and traumatic etiology have been identified as the possible clinical predictors of short-term consciousness recovery in patients with prolonged DoC (10).

On the other hand, only a few studies have investigated the clinical conditions in patients with prolonged DoC in the long term (8, 9, 11). As the evidence about functional and clinical conditions of these patients in the middle- and long-term is limited, a consensus about solid predictors of recovery is still missing.

More recently, a higher total score on the Coma Recovery Scale-revised (CRS-R) at admission in the intensive rehabilitation unit (IRU) and the progression of this score during the first 4 weeks of hospitalization have been identified as prognostic factors for consciousness or responsiveness recovery in the medium-term (12, 13). Moreover, for what concerns quantitative measures, the bilateral absence of cortical (N20) components of somatosensory-evoked potentials (SEPs) is considered the most accurate neurophysiological marker for an unfavorable prognosis of consciousness improvement with high specificity, especially in the postanoxic etiology of DoC, for both short- (14) and long-term prognoses (15, 16). Further, visual analysis of conventional EEG background activity and reactivity collected upon admission to the IRU was recently identified as a possible predictive marker for a better evolution of consciousness at discharge (17–19) or 6 months after brain injury (20). Additional factors have been reported to be prospectively associated with motor improvement in sABI patients who recovered consciousness: the evaluation of residual central neural motor circuit and the analysis of motor-evoked potential (MEP) may increase prognostic accuracy for motor limb recovery and thus improve the functional prognosis in brain-damaged patients (21). Further, the presence of critical illness neuromyopathy (CINMP), diagnosed by electromyography at the IRU admission, has been reported to associate with a lower functional outcome at discharge (18, 22).

Additionally, the influence of genetic and environmental (epigenetic) factors on the outcome of severe traumatic brain injuries (23) is also studied in the literature. New prognostic information was provided on acquired brain lesions by the analysis of different genes, such as those encoding apolipoprotein E (ApoE), catechol-O-methyltransferase (COMT), and brain-derived neurotrophic factors (BDNFs) (24). Expression of different ApoE protein isoforms (E2, E3, E4) affects lipoprotein complexes associated with LDL receptors. Based on this, ApoE is essential for the normal catabolism of triglyceride-rich lipoprotein constituents, and the different ApoE isoforms have a deep effect on peripheral lipid metabolism. ApoE, in the brain, acts as a scaffold with the formation of HDL particles, which promote the proteolytic degradation of soluble forms of amyloid-beta; therefore, ApoE may be of interest in understanding the impact of genetics on the long-term outcome of sABI patients (25). BDNF is a member of the nerve growth factor family synthesized by neurons and expressed by different brain regions, including the cortex and hippocampus, acting to modulate synaptic connections and to form new synaptic contacts. There is a functional single-nucleotide sequence polymorphism changing valine to methionine at codon 66 that has been associated with memory impairment in neurodegenerative disease (26). Given these premises, an accurate and comprehensive characterization of patients after sABI, with or without DoC, should include neuronal damage, potential neuroplasticity, and neurofunctional, and clinical status. A comprehensive assessment of individual patients' profiles and the identification of predictive markers could allow the planning of personalized rehabilitation and treatment pathways, based on solid prognostic information.

This paper describes the background and methods of an Italian multicenter study that will prospectively investigate outcomes and baseline predictors or biomarkers of functional recovery in a large cohort of patients with sABI admitted to postacute inpatient rehabilitation. The study aims to identify clinical and functional factors, neurophysiological patterns, and genetic polymorphisms which may predict short- and long-term functional recovery and, possibly, support the clinicians in optimizing rehabilitation outcomes by the development of personalized strategies. For this purpose, patients will be assessed at IRU admission, at 3 months from IRU admission, and in the chronic phase (i.e., until 24 months from the acute event).

## Methods and Analysis

### Study Design and Participants

The proposed study is a longitudinal multicenter observational cohort study, including four IRUs of the Fondazione Don Carlo Gnocchi Institute (FDG) located in Northern (Milano, La Spezia), Central (Firenze) and Southern Italy (Sant'Angelo dei Lombardi) of the Fondazione Don Carlo Gnocchi Institute (FDG). The study was registered on ClinicalTrials.gov with the following registration number: NCT04495192.

Patients with sABI admitted in the above-mentioned IRUs and fulfilling the following inclusion criteria will be consecutively enrolled.

Inclusion criteria were as follows:

- time from sABI < 4 months
- age >18 years
- written informed consent.

No additional exclusion criteria, other than the absence of a signed informed consent or age and time from onset out of range, will be considered.

The sample size estimation was based on the functional recovery assessed by GOS-E as the outcome. Based on the average number of sABI patients referring to the four IRUs involved in the study in the previous years, we estimate 36 months to enroll 520 patients.

In particular, UWS and MCS sample size was dimensioned separately building on the literature findings that show a favorable outcome [GOS-E>4, (4, 5)] in 3% and 47% of cases, respectively (27). Uncertainty of an additional 3–5% respectively on those values was considered. Assuming a level of significance of 0.05, a sample of 384 patients for the MCS group and 125 for the UWS group was achieved. Based on the average number of sABI patients referring to the four IRUs involved in the study in the previous years, the sample was assigned to each center (200 from Firenze and Milano, 60 from La Spezia and Sant'Angelo dei Lombardi).

### Data Collection

The multiprofessional staff (i.e., neurologists, neurophysiologists, physiatrists, internists, neuropsychologists, neurophysiological technicians, nutritionists, physiotherapists, speech therapists, occupational therapists, and nurses) of each center will gather clinical and neurophysiological data and structural indices of overall brain damage from brain imaging. To promote a training on specific datasets, the coordinating center will schedule meetings with all the involved users, before the starting of the enrollment, to guarantee homogeneity of data collection. For the analysis of genetic or epigenetic markers, in the presence of signed informed consent, a blood sample will be collected from each patient along with the routine blood drawn at T0, T1, and T2, frozen and later transported to the Neurogenetics Laboratory of Careggi Hospital, in Firenze.

**Figure 1:**
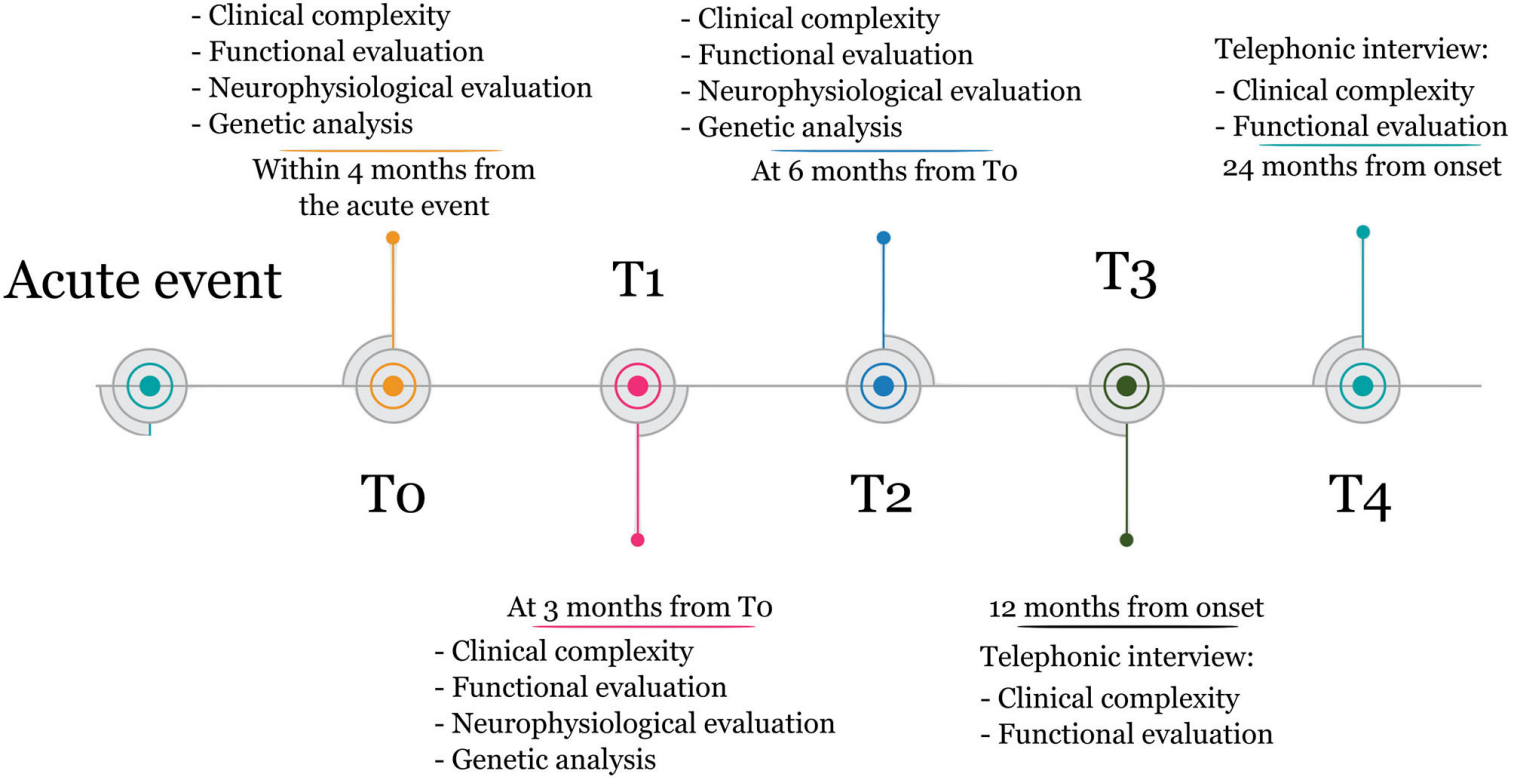
Study timeline.

Measures will be taken at (1) inpatient rehabilitation admission (T0), (2) 3 months from T0 (T1), (3) 6 months from T0 (T2), (4) 12 months from the acute event (T3), and (5) 24 months from the acute event (T4). The timeline of the study is illustrated in Figure 1.

#### Clinical Evaluation

At admission (T0), demographical and clinical data, including those related to the acute event, will be recorded. In particular, sABI etiology (posttraumatic, postanoxic, postischemic, posthemorrhagic or other), localization and extension of the brain damage, as described by brain imaging, the presence of clinical complications in the acute phase (e.g., a second brain event, acute epileptic event), treatment received during the acute phase (e.g., thrombolytic therapy, neurosurgery), and eventual procedural complications, will be recorded.

Markers of clinical and nursing complexity will be assessed at T0, T1, and T2, including the presence and severity of comorbidity as measured by the Cumulative Illness Rating Scales, the body mass index, the presence of medical devices, and pressure ulcers. At 3 months (T1) and 6 months (T2), any clinical event that occurred between the T0 and T1 and between T1 and T2, respectively, will be registered. More specifically, late epileptic event, recurrence, the presence of paroxysmal sympathetic hyperactivity (PSH), and the emergence of spasticity or heterotopic ossifications will be considered. Also, the pain will be assessed using the Nociception Coma Scale, the Numeric Rain Scale, or the pain assessment in advanced dementia scale, according to the patient's level of consciousness. Additionally, all pharmacological therapy introduced for epilepsy, PSH, spasticity, or pain, will be recorded. Past medical history, and baseline functional evaluation including measures of consciousness state, impairment and disability, administered by skilled members of the multiprofessional team, will be recorded at each time point, according to the patient's clinical state:

- Level of consciousness will be assessed using the Italian version of the CRS-R. The best score obtained from five administrations performed in seven days will be recorded. When available, the lowest Glasgow Coma Scale achieved during the acute phase will be recorded.
- The neurocognitive and behavioral assessment will include the anamnestic Cognitive Reserve Index, the level of cognitive functioning, the Galveston Orientation and Amnesia Test, the Apathy Evaluation Scale, the Agitated Behavior Scale, the Halpern communication scale or the Goodglass–Kaplan communication scale, the Aachener Aphasie Test, the Hospital Anxiety and Depression Scale or the Aphasic Depression Rating Scale, and the Phone Montreal Cognitive Assessment.
- The sensorimotor impairment will be assessed by the Trunk Control Test and the modified Ashworth Scale, whereas dysphagia will be assessed using the Functional Oral Intake Scale.
- Disability will be measured by the Early Rehabilitation Barthel Index, the Disability Rating Scale, the Functional Independence Measure, and the Glasgow Outcome Scale-expanded (GOS-E).

After hospital discharge, at T3 and T4, a phone interview with the patients and their principal caregiver will be carried out, to evaluate their clinical state including consciousness level, disability level, dysphagia, and participation level using the Community Integration Questionnaire. The quality of life as perceived by both the patient and the caregiver will be assessed through the quality of life (QoL) after the Brain Injury Scale (QoLibri). Additionally, any relevant clinical event occurring after discharge will be recorded.

All assessment tools are summarized in Table 1, and the details on the references for each tool are reported in Supplementary Table S1.

**Table 1:** Assessment tools.

**Area of competence**		**Evaluation tool**	**T0**		**T1**	**T2**	**T3**	**T4**
							**Phone follow-up**	
			**Anamnestic evaluation**	**Baseline evaluation**	**3 months from T0**	**6 months from T0**	**12 months from onset**	**24 months from onset**
		Etiology		X				
		Brain localization and extension		X				
		Trial of Org 10172 in Acute Stroke Treatment		X				
**Acute event**		Oxfordshire Community Stroke Project		X				
		Early clinical complications		X				
		Treatment received during the acute phase (thrombolysis/fibrinolysis)		X				
		Procedure complications		X				
		Cumulative Illness Rating Scale	X	X	X	X		
		Presence of medical device		X	X	X	X	X
**Clinical complexity**		Body mass index	X	X	X	X	X	X
		Braden scale		X	X	X		
		Relevant medical complications				X	X	
		Pain assessment (NRS/NCS/PAINAD)		X	X	X		
	**Consciousness**	Glasgow Coma Scale		X				
		Coma Recovery Scale revised		X	X	X	X	X
	**Neurocognitive evaluation**	Cognitive reserve index	X					
		Level of cognitive functioning		X	X	X		
**Functional assessment**		Galveston Orientation and Amnesia Test			X	X		
		Apathy Evaluation Scale			X	X		
		Agitated Behavior Scale			X	X		
		Halpern communication scale or the Goodglass–Kaplan communication scale			X	X		
		Montreal cognitive assessment					X	X
	**Sensorimotor assessment**	Trunk Control Test	X	X	X	X		
		Modified Ashworth scale		X	X	X		
		Functional Oral Intake Scale		X	X	X	X	X
	**Disability level**	Early rehabilitation Barthel Index	X	X	X	X		
		Disability Rating Scale		X	X	X		
		Functional improvement measure		X	X	X		
		Glasgow Outcome Scale-expanded	X	X	X	X	X	X
	**Participation level**	Community I Questionnaire					X	X
		Electroencephalography		X	X			
**Neurophysiological evaluation**		Somatosensitive Evoked potential		X				
		Motor-evoked potential		X	X			
		Electromyography		X	X			
		ApoE		X	X	X		
**Genetic markers**		Brain-derived neurotrophic factor		X	X	X		
		Dopamine receptor 2		X	X	X		

***Legend:** Clinical complications: metabolic, cardiovascular, muscular and skin, gastrointestinal, genitourinary, respiratory, epilepsy or myoclonus, neurosurgery compliances, POA, PHS*.

#### Neurophysiological Assessment

Neurophysiological evaluations will be performed within 1 week after the IRU admission. In case of the availability of SEP recorded in the acute–subacute setting, the examination will not be repeated.

##### EEG (At T0 and T1)

An EEG of 30 min of wakefulness will be performed using Galileo NT (EBNEUR) with 32 channels through an EEG prewired headcap with 19 recording electrodes, one ground electrode, and a reference positioned according to the 10–20 international system. The signal will be sampled at 128 Hz, and the sensitivity will be set at 7 μV/mm and then filtered (1.6–30 Hz). The examination will be carried out on a bed or a wheelchair based on the patient's clinical conditions. During the 30-min recording period, patient reactivity will be assessed through active or passive eyes opening and closing, depending on the degree of patient collaboration, and through acoustic and nociceptive stimuli, each stimulus repeated three times.

The EEGs will be classified according to the ACNS terminology (28); the EEG descriptors taken into account will be continuity, voltage, frequency, reorganization of an anterior or posterior gradient of the background activity, symmetry, the presence of spontaneous variability, the presence of reactivity of the background activity, and the presence of epileptic discharges and detectable EEG stage II sleep patterns. Additionally, epileptiform abnormalities will be classified as follows: (1) interictal epileptiform activity, (2) periodic discharges, and (3) electrographic seizures.

##### Somatosensory-Evoked Potential (T0)

Right and left median nerves will be stimulated at the wrist by a bipolar surface electrode, at an intensity of 4–5 mA, greater than the one needed to evoke a muscular response from abductor pollicis brevis, with a pulse duration of 0.2 ms and stimulus rate at 3 Hz. Recordingstainless steel needle electrodes will be placed at different locations to ensure recording of peripheral, spinal, bulbar, and cortical components. The locations to consider will be the following: Erb's point (referred to contralateral Erb's point), spinous process CV7 (referred to the anterior neck), and C3 and C4 (referred to Fz and ipsilateral mastoid). At least two repetitions (averages of 300 responses) will be acquired to assess the reproducibility of waveforms. The analysis time will be 100 ms, with a bandwidth of 5 Hz−3 kHz. The N20 wave will be identified as the major negative peak with a latency of approximately 20 ms from the stimulus, whereas P25 will be identified as the major positive peak following N20. Considering the cortical responses of each hemisphere, we will obtain six SEP patterns: *NN-NP-PP-NA-AP-AA*. *N* will stand for normal (N20/P25 amplitude is normal); *P* will stand for pathological (N20/P25 amplitude <1.2 μV); *A* will stand for N20 absence (if no reproducible components will be identified in the presence of a cervical potential) (29).

##### Motor-Evoked Potentials (T0)

Transcranial magnetic stimulation (TMS) will be performed according to the standard criteria of the International Federation of Clinical Neurophysiology (30). Motor-evoked potentials (MEPs) will be recorded from abductor digiti minimi (ADM) and tibialis anterior (TA) muscles. Exclusion criteria are (1) the presence of pacemaker, (2) the presence of physical limitations (e.g., bandages or plasters) that prevent the evaluation of peripheral muscular response through the compound muscle action potential (CMAP), and (3) the presence of craniotomy.

The examination will be conducted with the Medelec Synergy electromyograph (Natus Europe) associated with the MagVenture MagPro Compact magnetic stimulator equipped with a circular coil. Disposable surface electrodes (BionenFlorence, Italy) will be placed on the muscle under examination. Once the motor response of supramaximal amplitude will be obtained, the magnetic stimulus will be delivered. A circular coil will be used to map the area of interest corresponding to the target muscles studied. For the TA, given the cortical representation, the coil will be positioned slightly ipsilateral to the side under examination in correspondence to Cz, whereas for the ADM, the coil will be positioned contralateral, taking Cz as reference. The stimulus will be delivered in a resting situation because of the low level of patient consciousness. A stimulus will be provided at the paraspinal level only in the assessment of the upper limbs due to the increased accessibility of the stimulation site.

The MEP will be classified as follows:

- Normal.
- Pathological if the MEP will be recordable but not within the reference values for amplitude and/or absolute latencies.
- Absent if the potential amplitude will be <50 μV could be obtained after 5 stimuli at 100% intensity.

##### Electroneurography/Electromyography (T0)

The electroneurography/electromyography (ENG/EMG) will be performed using a MEDELEC Synergy Oxford device. All patients will undergo conventional orthodromic motor and antidromic sensory nerve conduction studies on eight motor nerves (axillary, ulnar, common peroneal, and tibial nerves, bilaterally) and four sensory nerves (ulnar and sural nerves, bilaterally). The muscular activity will be assessed with concentric needle electrodes at rest and, when possible, during contraction. Sensory nerve action potential (SNAP), distal motor latencies, CMAP, and nerve conduction velocities will be registered. Spontaneous activity and, when possible, recruitment and interference patterns will be detected bilaterally by needle EMG from deltoid, ADM, and tibial anterior muscles. Patients with conduction velocities <20% of the lower limit will be diagnosed as having possible polyneuropathy of other causes and will be excluded from the analysis.

For conduction velocities, normal limits will be defined as mean ± 2 standard deviations (SDs) of normative data of our laboratory. For CMAP and SNAP, the lower limit will be set to the 5th percentile derived from the normative data of our laboratory (31).

#### Analysis of Genetic Marker

For the subgroup of patients who will sign a further, dedicated informed consent, a blood sample, along with the routine blood drawn at T0, T1, and T2, will be frozen and sent to the University Hospital Neurogenetic Lab of Florence.

Genomic DNA will be obtained from EDTA-whole venous blood samples by Automated Systems QiaCube (Qiagen). The genetic analysis of five different single-nucleotide polymorphisms on APOE and BDNF will be performed by high-resolution melt (HRM) analyses and direct sequencing on automatic genetic analyzer. ApoE genotypes will be investigated by HRM with two sets of PCR primers designed to amplify the regions encompassing rs7412 [NC_000019.9: g.45412079C>T] and rs429358 (NC_000019.9: g.45411941T>C). The samples with known ApoE genotypes, which have been validated by DNA sequencing, will be used as standard references. Analysis of DNA methylation (DNAm) levels of BDNF will be performed according to the following protocol: equal amounts of a genomic DNA sample will be put in four separate tubes into which buffer and the appropriate restriction enzyme combinations added to detect different methylated DNA fractions. The product of a mock digest (Mo) contains all of the input genomic DNA. The product of the methylation-sensitive restriction enzyme mixture (enzyme A) digest (Ms) contains methylated DNA sequences, whereas the product of the methylation-dependent restriction enzyme mixture (enzyme B) digest (Md) contains unmethylated DNA sequences. The product of a double digest (Msd) measures the background and the success of both enzymatic digestions. To analyze the amount of DNA in each digest of each sample and determine the methylation status of CpG islands in gene promoters, all the aliquots will be amplified by a real-time PCR (Rotor-Gene 6000 Corbett Research). The analysis of the BDNF rs6265 will be performed by HRM using the following PCR primers 5′- ACTCTGGAGAGCGTGAATGG-3′ and 5′-ACTACTGAGCATCACCCTGGA-3′. The three genotypes will be identified through sequencing (SEQStudio Automatic Genetic Analyzer Life Technologies). The methylation levels of the gene promoters will be analyzed by the Epitec II DNA Methylation Enzyme Kit (QIAGEN). The genetic polymorphism data will be compared to sex- and age-matched controls of the DNA banking of the neurogenetic laboratory.

### Rehabilitation

The rehabilitation intervention is defined in an ICP based on the International Classification of Functioning, Disability, and Health-World Health Organization (ICF 2001, WHO) model of functioning (32). Synthetically, the standardized rehabilitation assessment and process of care provide, according to the national requirements, at least 3 h per day of specific rehabilitation including physiotherapy, neuropsychological therapy, speech and dysphagia therapy, and occupational therapy. Based on systematic screening at admission, weekly team revisions of the individual rehabilitation plan, and emerging needs during the rehabilitation stay, it is determined the type of aid the patients may need. Additionally, specific training sessions are planned for the patients to be familiar with the prescribed aids. When indicated, psychological support to the patient and/or family is also provided. Physiotherapy may also include robotic rehabilitation of the upper limb according to the individual rehabilitation plan defined by the interdisciplinary team. Specific rehabilitative interventions are summarized in Supplementary Table S2.

### Outcome Measures

The primary outcome is the functional recovery, given by the achievement of a moderate functional disability (GOS-E>4), whereas the independent variables will be the severity of the patients' clinical state at admission. For those patients who will remain in DoC at discharge from the IRU, given that the GOS-E does not allow accurate discrimination between different states of consciousness, consciousness improvement will be considered. The improvement will be stated as the transition from one state of consciousness to a higher one (based on the CRS-R assessment). These outcomes will be measured both at short-term evaluations, T1 and T2, and at long-term ones, T3 and T4.

Secondary outcomes will be considered at the same time points. They are (1) tracheostomy decannulation success and its timing and (2) complete oral feeding recovery corresponding to FOIS score ≥4. Additionally, at follow-up time points (T3 and T4), the level of participation, using the CIQ scale, and the subjective and caregiver perceived QoL, using the QoLibri, will also be considered as secondary outcomes.

### Data Collection and Management

Data collection will be carried anonymously on REDCap, an online-based software for the design of databases. This will allow for higher quality and robustness of the data collected and a reduction of missing data.

Data will be collected in a pseudo-anonymized way, attributing a record ID to each patient on the electronic database and saving the correspondences between names and identification codes on a separate document that will be destroyed at the end of the study.

### Statistical Analysis

Statistical analyses will be performed using SPSS 27.0 software (IBM SPSS, Chicago, Illinois, USA). Descriptive statistics of variables belonging to the four clinical assessment categories (clinical and nursing complexity, neurological profile, functional evaluation and neurophysiological parameters, and methylation levels of the BDNF gene) will be provided for each time point. The statistics will include absolute counts and relative frequencies for categorical and dichotomous variables, whereas numeric variables will be reported as mean and SD or median and interquartile range, according to the normal or nonnormal distribution. The violation of the normality assumption will be ascertained using the Shapiro–Wilk test.

Variable comparisons at two-time points will be performed by paired *t*-test or Wilcoxon signed-rank test for numeric scales, as appropriate for the normality of the data, and by the McNemar test for paired categorical or dichotomous data. For those variables evaluated at all time points, differences over time will be ascertained using a repeated measures ANOVA (Bonferroni and Sheffé corrections will be applied to multiple comparisons) or a Friedman test, for continuous variables, and a Cochran's *Q* test for dichotomous variables. In all the above-mentioned analyses, a *p*-value <0.05 will be considered as statistically significant.

For what concerns the primary outcome, those variables identified through univariate analysis as recovery predictors on the GOS-E scale will be grouped in categories based on the nature of variables: clinical or nursing complexity (including anagraphical data), neurological profile, functional factors, neurophysiological parameters, and genetic profile. For each category, a multiple logistic regression analysis will be conducted to investigate whether there is a relationship with the primary outcome. The analysis will be then repeated including variables from different categories that showed a significant association with the functional recovery at discharge. For the secondary outcomes, a two-step process constituted on univariate analyses adjusted by age and sex and multivariate analysis will be performed. More in detail, univariate analyses, adjusted by proper confounders, and multivariate analyses will be performed using logistic regressions.

A further step will involve advanced machine learning methods for the short- and long-term prediction of outcomes. Classical solutions such as linear and logistic regression will be compared with solutions based on support vector machines, random forests, or multilayer perceptrons and also with “deep” artificial neural networks (convolutional neural networks, recurrent neural networks, and ensemble learning solutions). The performances of different algorithms in predicting recovery outcomes will be compared in terms of accuracy, F1-score, root mean square error, and determination coefficient. The effect of hyperparameter tuning and automatic features selection strategies will also be tested by nested crossvalidation, with the final aim to quantify the solution generalization capability when applied to new patients who were not included in model definition and training.

To assess the clinical applicability of these solutions, the prediction of recovery milestones by automatic methodologies will be compared to the evaluation of a multidisciplinary team, which will be kept blind with respect to the algorithm outputs. This assessment is usually performed in clinical practice approximately 2 weeks from admission to inform the caregivers of the patients' expected recovery path. Opinions by both single professionals and the full consensus will be compared to the automatic tool predictions.

## Discussion

In the last decades, the number of survivors of sABI, including those with DoC, has significantly increased due to considerable advances in intensive care technology and neurosurgical procedures. After the acute phase, patients usually have access to the in-patient IRU, to exploit their recovery potential: the IRU has indeed replaced the ICUs becoming the new “turning point” for the sABI patients. Unlike postanoxic sABI patients, for whom solid predictors are usually recorded in the acute phase (29), in sABI patients with other etiologies, the lack of predictive indices of the acute phase justifies the research shift toward rehabilitation settings. The IRU is the starting point of a long and complex rehabilitation path that does not always lead to a reintegration of the patient into his/her home environment, especially in those patients who remain in a state of DoC. Although rehabilitation is highly recommended after sABI (33), the heterogeneity of the etiologies and underlying brain damage mechanisms makes the development of standardized rehabilitation pathways highly challenging. Different treatment approaches have been proposed in the past two decades, using both conventional and new technologies (34), and few studies addressed the benefit of early rehabilitation in DoC (35, 36) but no randomized controlled trial has been published in this field. Also, health systems coordinating sABI rehabilitation delivery, outcome assessment, and also resources for sABI care and rehabilitation, are still extremely variable among geographic regions worldwide (37, 38). In Italy, sABI rehabilitation recommendations are a general reference to national guidelines (2, 39), but their operational definition into protocols and pathways is very heterogeneous and significantly affected by the different standards applied at regional and even local levels, creating a high risk for suboptimal care (40). Given the high variability of patients' features and responses, the customization of rehabilitation treatment according to biomarkers and predictive factors has great potential for the optimization of rehabilitation processes and outcomes.

The recovery of consciousness for patients with a DoC is the first rehabilitative goal and one that largely conditions subsequent objectives. Identifying the prognostic factors of consciousness improvement needs a correct diagnosis of consciousness upon admission to the IRU. More and more literatures were provided in the last few years to support diagnostic decisions on DoCs, but the evidence remains insufficient, given the complexity and the ethical weight of the issue. The CRS-R scale has been recommended by the Congress of Rehabilitation Medicine for the clinical assessment of consciousness levels in patients with DoC (41). However, several potential confounding factors related to examiner, patient, and environment could hamper the clinical diagnosis, and it has been estimated that up to 40% of noncommunicating patients with DoC may be wrongly classified (42). Along with the fluctuation of the consciousness level itself, some concomitant clinical states, such as aphasia, apraxia, object, or visual agnosia, marked spasticity (43), and hyper or hypotonus was reported to produce impossible or improbable CRS-R scores (44). Additionally, some associated pathologies or clinical conditions, such as the presence of a CINMP (22) or the presence of tracheostomy (45, 46), may limit the recognition of voluntary behavior and, consequently, hinder a correct diagnosis of consciousness (42). This risk of misdiagnosis has serious ethical concerns and deep implications for medical management and the decisions of the patient' families (47). For the same purpose, sophisticated diagnostic techniques such as functional magnetic resonance imaging (fMRI) and positron emission tomography (PET) have been developed (48). By detecting volitional activity, not recognizable on clinical grounds, they provide a more robust evaluation of consciousness, having, in addition, the advantage to provide useful prognostic information. Besides these technologically advanced tools, which are unsuitable for large-scale use, since they are sophisticated, expensive, and time-consuming, some studies investigated the diagnostic and prognostic value of widely used and easily repeatable instrumental approaches (e.g., standard clinical EEG, ENG/EMG, or evoked potentials) (15, 18, 19). Both the European Academy of Neurology and the American Academy of Neurology have recently recommended the use of a multimodal evaluation by combining clinical evaluation, EEG, SEP, and functional neuroimaging for improving clinical classification and prognostication of people with DoC (49, 50). However, a broad consensus on diagnostic and prognostic procedures for the clinical care of individuals with prolonged DoC has not been reached, yet. Indeed, a recent international survey showed that diagnostic and prognostic procedures for DoC are still extremely variable across geographic regions worldwide (37). In the present work, for greater accuracy of diagnosis, it was planned to perform a multimodal consciousness diagnosis by the integration of clinical evaluation (through the CRS-R) with specific EEG patterns related to consciousness (19).

After regaining consciousness, patients with sABI often present many factors that may limit their recovery. Indeed, significant neurological impairments, including motor deficits, myoclonus, dystonia, movement disorders, aphasia, neglect, abulia, impairments in attention, memory, executive functions, mood disorders, and epileptic seizures, may have a dramatic impact on the level of functional independence and quality of life (51). Also, sABI patients frequently present a high clinical complexity, with a high rate of comorbidities (6) and medical complications (7) that can further compromise the functional prognosis. For their complex nature, patients with sequelae of sABI require several assessment instruments to correctly quantify every residual symptom and adequately reflect their clinical state during the acute, postacute, and community-living sABI phase. Furthermore, given the long duration of the rehabilitation process and the continuous evolution of patients over time, the measurement of outcomes also needs to be dynamic, to accurately depict, as realistically as possible, the patient's situation at each time point. Recently, the Italian Society of Physical and Rehabilitation Medicine proposed a minimum assessment protocol for post-sABI patients (52); however, there is no National Health System (NHS) requirement as to the measures of sABI rehabilitation outcomes. In this study, evaluation tools were chosen in accordance with the Italian Society of Physical and Rehabilitation Medicine minimum assessment protocol (52), and targeted outcome measures were hierarchically organized based on the patient's state of consciousness. Only after the consciousness recovery, some measures of functioning, including motor and cognitive impairment, activity, and participation were applied. Further, special attention was given to the care burden (the presence of compliances and comorbidities, the presence of medical devices, oral/artificial feeding, and communication capabilities) to assess the impact, on both the caregiver and the NHS, at discharge and until 24 months after the acute event.

The personalization of rehabilitation strategies is yet largely bounded by the limited knowledge existing on biomarkers of rehabilitation outcome. Thus, besides the clinical–functional component, this study will also investigate neurophysiological and genetic parameters as possible biomarkers of functional outcome. In addition to neurophysiological examinations, to improve the diagnosis of causes of impaired consciousness, the former can make a valuable contribution in assessing subsequent outcomes such as functional autonomy (including both motor and cognitive influence) and the degree of participation. As to motor recovery, it will be verified whether and how the combination of the collected neurophysiological measures (ENG/EMG and MEP) could contribute to the motor prognosis. Recent studies have emphasized the importance of some neurophysiological markers as possible predictors of motor recovery after vascular brain injuries (21). Namely, an ipsilesional loss of power in the alpha frequency band and an increase in the delta frequency band in the EEG, detected within 2 weeks of brain vascular damage, are linked to a poor outcome (53). Moreover, the presence of CIPNM in sABI patients was found to be related to reduced functional recovery and longer time to tracheostomy decannulation (22). The EEG, in addition to the identification of epileptic abnormalities as a possible predictive index of the remote structural epilepsy (SE) occurrence, could provide data for quantitative analysis (connectome) for the eventual prediction of SE onset during the IRU stay and after it. Indeed, the prevalence of SE after sABI was estimated at 16.4% (54), and posttraumatic sABI patients are more likely to develop SE (51). The SE could hamper recovery of consciousness in patients with prolonged DoC (55) and increase mortality in patients with vascular sABI (56).

Regarding genetic factors, a significant association of ApoE polymorphism was found with poor recovery from a posttraumatic coma (57) and, lately, with the diagnosis of prolonged UWS (58). In some studies, E4 carriers have shown longer recovery and poor outcomes by 6 months following traumatic brain injuries (7, 8). In this study, the ApoE genotype will be investigated to explore its possible effect on the functional (including motor and cognitive aspects) and participation outcomes in sABI survivors. As for BDNF polymorphism, many studies analyzed the interaction of the V66M in brain injury, with contrasting findings (59, 60). However, BDNF gene expression is complex, and nine functional promoters confer tissue and brain region-specific expression (61). For that reason, it is crucial to explore methylation levels of BDNF to analyze its expression. COMT is one of the several enzymes that degrade catecholaminergic neurotransmitters. Catecholamines are thought to play a role in recovery and functioning after traumatic brain injury (62) and variation in the metabolism of catecholamines after injury may influence cognitive function and outcome (23). Because of the biological implications of the COMT, genetic variation (methionine to valine substitution at codon 158 leads to differing enzyme activity) on catecholamine metabolism genotypes associated with lower or higher levels of catecholamines will be examined concerning the sABI recovery. Many different clinical outcomes following sABI could be influenced by these genetic factors or modulated by environmental factors that could alter gene expression by epigenetic mechanisms, such as DNA methylation (DNAm). These changes may be rapid and dynamic, and although the field of epigenetics is now well established, interest in the epigenetic mechanisms involved in sABI pathophysiology and outcome has only recently emerged (63). Moreover, knowledge of the role of epigenetic changes associated with neural plasticity, learning, and memory reinforces the idea that these mechanisms could influence the consequences of brain injury, the recovery and the response to therapies, providing innovative approaches to sABI recovery and rehabilitation. In this study, it will be analyzed if there is a possible correlation between methylation levels of BDNF and DNA damage-inducible (Gadd) 45 protein (GADD45) genes and clinical outcome after sABI. GADD45 proteins act through a variety of molecular signaling cascades, including the cell-cycle control mechanisms, histone regulation, and epigenetic DNA demethylation. It will be of interest to evaluate methylation levels of these two genes in the recruited patients at the time of admission and 3 and 6 months after the event. Understanding the influence of individual epigenetic patterns on the intensive rehabilitation functioning after sABI may open new perspectives on the rehabilitation pathways.

To identify a profile of sABI patients based on a multiparameter assessment, the combination of the many recorded measures can be best exploited by machine learning analysis. The applicability of these solutions is also supported by the consideration that, with the only exception of the neurogenetic assessment, all the clinical and instrumental variables selected in this study are easily exportable to other clinical realities because of their low cost and high feasibility. Specifically, in this context, an accurate prediction is relevant both for planning a personalized rehabilitation pathway and for communication with relatives and caregivers. For these reasons, the ultimate goal of this study is to reliably predict the main recovery milestones, including the achievement of a consciousness recovery, tracheostomy decannulation, complete oral nutrition, functional independence, and the recovery of participation in familiar and social contexts. Differently from what is most often found in the existing literature, the aim of these analyses will be two-fold, addressing both the success in the achievement of these goals and their timing. The candidate predictors of these solutions will be selected from a comprehensive evaluation of sABI patients, including the biomarkers obtained from genetic sequencing and quantitative and qualitative EEG features.

Finally, another innovative point will be the comparison of these models with the clinical assessment performed by a multidisciplinary team within the first 2 weeks from admission. This comparison will represent a first step in the direction of computational tools translation into clinical practice.

In conclusion, this study aims to identify reliable prognostic markers of consciousness recovery, functional autonomy, and participation level in the late-acute and chronic phase after sABI. In addition to the classical multivariate logistic regression analysis, advanced machine learning algorithms will be crossvalidated to obtain data-driven prediction models and allow stratification into subgroups of patients with a high or low probability of recovery. The development of such predictive models aims at helping clinicians and patients' families to plan personalized treatment strategies in terms of intensity, duration, and protocols of a comprehensive rehabilitation program and to make appropriate decisions regarding treatment and care. Finally, it should be noted that patient profiling will include measures and procedures that are easily available at the patient's bedside, with affordable time, resources, and money to determine a real impact on the NHS and to optimize the use of resources.

## Ethics Statement

The studies involving human participants were reviewed and approved by Ethics Committees (Firenze: 16606_OSS, Milano: 21/2020/CE_FdG/FC/SA; Sant'Angelo Dei Lombardi: 1560; La Spezia: pending decision). The patients/participants provided their written informed consent to participate in this study.

## Author Contributions

BH, CM, and FC were involved in the general study design and contribution to the whole manuscript draft. BH, FC, AG, and BN carried out the manuscript draft. FDr, DM, JN, EP, and VC contributed in the design of functional assessment protocol. AR, ID, AC, and AE contributed in the design of the neurological assessment protocol. AG, MS, AMai, RS, and TA contributed in the design of the neurophysiological assessment protocol. BN, SB, and AI performed the design of the neurogenetic assessment protocol. AMan and SC will be involved in the statistical analyses. CC contributed in the draft revision. All author contributed to the article and approved the submitted version.

## Funding

This study was funded by RICERCA CORRENTE 2020 from IRCCS Don Gnocchi.

## Conflict of Interest

The authors declare that the research was conducted in the absence of any commercial or financial relationships that could be construed as a potential conflict of interest.

## Publisher's Note

All claims expressed in this article are solely those of the authors and do not necessarily represent those of their affiliated organizations, or those of the publisher, the editors and the reviewers. Any product that may be evaluated in this article, or claim that may be made by its manufacturer, is not guaranteed or endorsed by the publisher.
