# Supplementary material for: Clinical, Neurophysiological, and Genetic Predictors of Recovery in Patients With Severe Acquired Brain Injuries (PRABI): A Study Protocol for a Longitudinal Observational Study
Source: Front Neurol. 2022 Feb 28;13:711312. doi: 10.3389/fneur.2022.711312 (PMC8919857; doi:10.3389/fneur.2022.711312)
Supplement: Supplementary file 1 [file Table_1.pdf]

Area of competence	*Evaluation tool*	*Acronym*	*Reference*
Acute event	Trial of Org 10172 in Acute Stroke Treatment	TOAST	Adams HP, Bendixen BH, Kappelle LJ, Biller J, Love BB, Gordon DL, et al. Classification of subtype of acute is-chemic stroke. Definitions for use in a multicenter clinical trial. TOAST. Trial of Org 10172 in Acute Stroke Treatment. Stroke. gennaio 1993;24(1):35–41.
	Oxfordshire Community Stroke Project	OCSP	Anderson CS, Taylor BV, Hankey GJ, Stewart-Wynne EG, Jamrozik KD. Validation of a clinical classification for subtypes of acute cerebral infarction. Journal of Neurology, Neurosurgery & Psychiatry. 1 ottobre 1994;57(10):1173–9.
Clinical complexity	Cumulative Illness Rating Scale	CIRS	Linn BS, Linn MW, Gurel L. CUMULATIVE ILLNESS RATING SCALE. Journal of the American Geriatrics Soci-ety. maggio 1968;16(5):622–6.
	Braden scale		Brown SJ. The Braden Scale. A review of the research evidence. Orthop Nurs. 2004 Jan-Feb;23(1):30-8. doi: 10.1097/00006416-200401000-00010. PMID: 14999950.
	Paroxistic Sympathic Hyperactivity	PSH	Baguley IJ, Perkes IE, Ortega JFF., Rabinstein AA, Dolce G, and. Hendricks HT. Paroxysmal Sympathetic Hyperactivity after Acquired Brain Injury: Consensus on Conceptual Definition, Nomenclature, and Diagnostic Criteria. Journal of Neurotrauma 2014, 31:1515– 1520

		Numeric Rain Scale	NRS	High AS, Macgregor AJ, Tomlinson GE, Salkouskis PM. A gnathodynanometer as an objective means of pain as-sessment following wisdom tooth removal. British Journal of Oral and Maxillofacial Surgery. agosto 1988;26(4):284–91.
		Pain assessment in advanced dementia	PAINAD	Costardi D, Rozzini L, Costanzi C, Ghianda D, Franzoni S, Padovani A, et al. The Italian version of the pain as-sessment in advanced dementia (PAINAD) scale. Archives of Gerontology and Geriatrics. marzo 2007;44(2):175–80.
		Nociceptive Coma Scale	NCS	Schnakers C, Chatelle C, Vanhau- denhuyse A, Majerus S, Ledoux D, Boly M, Bruno MA, Boveroux P, Demertzi A, Moonen G.,Laureys S. The Nociception Coma Scale: A new tool to assess nociception in disorders of con-sciousness. Pain 2010;148(2):215-9
Functional assessment	**Consciousness**	Glasgow Coma Scale	GCS	Teasdale G, Jennett B. Assessment of coma and impaired consciousness. A practical scale. Lancet 1974; 2: 81–84
		Coma Recovery Scale Revised	CRS-R	Estraneo A, Moretta P, De Tanti A, Gatta G, Giacino JT, Trojano L; Italian Crs-R Multicentre Validation Group. An Italian multicentre validation study of the coma recovery scale-revised. Eur J Phys Rehabil Med. 2015 Oct;51(5):627-34. Epub 2014 Mar 6
	**Neurocognitive evaluation**	Cognitive Reserve Index	CRI	Nucci M, Mapelli D, Mondini S. Cognitive Reserve Index questionnaire (CRIq): a new instrument for measuring cognitive reserve. Aging Clin Exp Res. 2012 Jun;24(3):218-26.
		Level of Cognitive Functioning	LCF	Hagen C, Malkmus D, Durham P. Cognitive assessment and goal setting. In Rehabilitation of the head injured adult: comprehensive management. Downey Ca: professional staff association of Rancho Los Amigos Hospital, Inc., 1979
		Galveston Orientation and Amnesia Test	GOAT	Bode RK, Heinemann AW, Semik P. Measurement properties of the Galveston Orientation and Amnesia Test (GOAT) and improvement patterns during inpatient rehabilitation. J Head Trauma Rehabil 2000, 15(1):637-55
		Apathy Evaluation Scale	AES	Lee B, Gleason C, Umucu E. Clinical utility and psychometric properties of the Apathy Evaluation Scale. Rehabil Psychol. 2020 Aug;65(3):311-312.
		Agitated Behaviour Scale	ABS	Bogner J, Corrigan J, Stange M, Rabold D. Reliability of the Agitated Behavior Scale. Journal of Head Trauma Rehabiltiation 1999;14:91–96.

		The Goodglass-Kaplan communication scale		Goodglass, H. and Kaplan, E. (1972) The Assessment of Aphasia and Related Disorders. Lea & Febiger, Philadelphia.
		Aachener Aphasie Test	AAT	Spaccavento S, Cafforio E, Cellamare F, Colucci A, Di Palma A, Falcone R, Craca A, Loverre A, Nardulli R, Glueckauf RL. Italian adaptation of the functional outcome questionnaire - aphasia: initial psychometric evaluation. Disabil Rehabil. 2018 Dec;40(24):2925-2930. doi: 10.1080/09638288.2017.1362042. Epub 2017 Aug 4. PMID: 28776480.
		Hospital Anxiety and Depression Scale	HADS	Snaith RP. The Hospital Anxiety And Depression Scale. Health Qual Life Outcomes. 2003;1:29. Published 2003 Aug 1. doi:10.1186/1477-7525-1-29
		Aphasic depression rating scale	ADRS	Benaim C, Cailly B, Perennou D, Pelissier J. Validation of the aphasic depression rating scale. Stroke. 2004 Jul;35(7):1692-6. doi: 10.1161/01.STR.0000130591.95710.20. Epub 2004 May 13. PMID: 15143288.
		Phone Montreal Cognitive Assessment	MoCA	Wong A, Nyenhuis D, Black SE, Law LS, Lo ES, Kwan PW, Au L, Chan AY, Wong LK, Nasreddine Z, Mok V. Montreal Cognitive Assessment 5-minute protocol is a brief, valid, reliable, and feasible cognitive screen for telephone administration. Stroke. 2015 Apr;46(4):1059-64. doi: 10.1161/STROKEAHA.114.007253. Epub 2015 Feb 19. PMID: 25700290; PMCID: PMC4373962.
	**Sensorimotor**	Trunk Control Test	TCT	Franchignoni FP, Tesio L, Ricupero C, Martino MT. Trunk Control Test as an Early Predictor of Stroke Rehabili-tation Outcome. Stroke. luglio 1997;28(7):1382–5.
		modified Ashworth Scale	mAS	Blackburn M, van Vliet P, Mockett SP. Reliability of Measurements Obtained With the Modified Ashworth Scale in the Lower Extremities of People With Stroke. Physical Therapy. 1 gennaio 2002;82(1):25–34.

	**assessment**	Functional Oral Intake Scale	FOIS	Battel I, Calvo I, Walshe M. Cross-Cultural Validation of the Italian Version of the Functional Oral Intake Scale. Folia Phoniatr Logop. 2018;70(3-4):117-123. doi: 10.1159/000490792. Epub 2018 Aug 8. PMID: 30089299.
	**Disability level**	Early rehabilitation Barthel Index	ERBI	Schonle PW. The Early Rehabilitation Barthel Index (ERBI) – an early rehabilitation focused extension of the Bar- thel Index. Rehabilitation 1995, 34: 69- 73
		Disability Rating Scale	DRS	Gouvier WD, Blanton PD, La Porte KK et al. Reliability and Validity of the Disability Rating Scale and the Levels of Cognitive Functioning Scale in monitoring recovery from severe head injury. Archives of Physical Medicine and Rehabilitation 1987, 68: 94-97
		Functional Improvement Measure	FIM	Hall KM, Mann N, High W et al. Functional measures after traumatic brain injury: ceiling effects of FIM, FIM+FAM, DRS and CIQ Journal of Head Trauma Rehabilitation, 1996, 11: 27-39
		Glasgow Outcome Scale Expanded	GOS-E	Wilson JT, Pettigrew LE, Teasdale GM. Structured interviews for the GOS and the GOS -E: guidelines for their use Jour- nal of Neurotrauma 1998 :15 573-585
	**Participation level**	Community I Questionnaire	CIQ	Lombardi F, Orsi P, Mammi P et al. Validità del Community Integration Questionnaire (CIQ) e dati normativi per l'Italia. Giornale Italiano di Medicina Riabilitativa, 1997, 11: 23-34
		Quality of life after brain injury	QoLibri	Formisano R, Silvestro D, Azicnuda E, Longo E, Barba C, Rigon J, D'Ippolito M, Giustini M, Bivona U. Quality of life after brain injury (QOLIBRI): Italian validation of the proxy version. Intern Emerg Med. 2017 Mar;12(2):187-198. doi: 10.1007/s11739-016-1536-1. Epub 2016 Sep 29. PMID: 27686362.

Legend: Clinical complications: metabolic, Cardiovascular, Muscular and skin, Gastrointestinal, genitourinary, Respiratory, epilepsy or myoclonus, neurosurgery compliances, POA, PHS

Table SM1: Supplementary material, Evaluation tools references
