# Supplementary material for: Clinical, Neurophysiological, and Genetic Predictors of Recovery in Patients With Severe Acquired Brain Injuries (PRABI): A Study Protocol for a Longitudinal Observational Study
Source: Front Neurol. 2022 Feb 28;13:711312. doi: 10.3389/fneur.2022.711312 (PMC8919857; doi:10.3389/fneur.2022.711312)
Supplement: Supplementary file 2 [file Table_2.pdf]

	LCF=1-3	*LCF=4-6*
MOTOR REHABILITATION	INDIVIDUAL NEUROMOTOR PHYSIOTHERAPY<br>• To improve surveillance and interaction with the environment<br>• To improve or maintain the joint flexibility<br>• To prevent spastic Hypertone<br>• To improve respiratory dynamics by strengthening the inspiratory and expiratory muscles to promote bronchial secretion management<br>• To check orthostatic hypotension and neurovegetative reactions during sitting position and verticalization<br>• To monitor Pain using the Nociceptive Coma Scale<br>• To inhibit reflexes and pathological postures	INDIVIDUAL NEUROMOTOR PHYSIOTHERAPY<br>• To promote neurobehavioral alterations control (state of psychomotor agitation, apathy, disinhibition)<br>• To improve orientation in time and space<br>• To improve focal attention<br>• To improve or maintain the joint flexibility<br>• To enhance the residual mobility<br>• To prevent Spastic Hypertone<br>• To improve respiratory dynamics by strengthening the inspiratory and expiratory muscles to promote tracheobronchial secretion management<br>• To check orthostatic hypotension and neurovegetative reactions during sitting position and verticalization<br>• To monitor Pain using the Nociceptive Coma Scale<br>• To prevent Musculotendon retraction through Passive Mobilization<br>• To improve balance and posture and postural responses in sitting and orthostatic position<br>• To improve trunk control and coordination in simple movements<br>• To reduce the Neglet Syndrome using the mirror therapy
	LCF=1-3	*LCF=4-6*
SPEACH THERAPY	• To improve surveillance and interaction with the environment<br>• To improve swallowing dynamics by promoting oral nutrition<br>• To improve the ability to understand verbal /non-verbal messages<br>• To improve management of salivary and tracheobronchial secretions<br>• To inhibit pathological oral reflexes	• To improve swallowing dynamics by promoting oral nutrition with a safe semi-solid/soft diet<br>• To improve the ability to understand verbal/non-verbal messages<br>• To improve management of salivary and tracheobronchial secretions<br>• To evaluate and manage the weaning path of the tracheostomic cannula<br>• To improve the ability to understand verbal/non-verbal messages<br>• To improve visuospatial perception<br>• To involve the rehabilitation team, the family members and/or caregivers in the observation and stimulation of swallowing and communication functions
Neurocognitive treatment when LCF>5 and anterograde amnesia resolution		• To evaluate the cognitive profile through appropriated cognitive batteries as needed<br>• To personify neurocognitive treatment as needed

Legend: LCF: Level of Cognitive Functioning

Table SM2: Supplementary material, Rehabilitative treatment
